# Comparing Individual and Community-level Characteristics of People with Ground Beef-associated Salmonellosis and Other Ground Beef Eaters: A Case-control Analysis

**DOI:** 10.1016/j.jfp.2024.100303

**Published:** 2024-05-23

**Authors:** Zainab Salah, Michelle Canning, David Rickless, Carey Devine, Ryan Buckman, Daniel C. Payne, Katherine E. Marshall

**Affiliations:** 1 Division of Foodborne, Waterborne, and Environmental Diseases, Centers for Disease Control and Prevention, Atlanta, Georgia, USA; 2 Oak Ridge Institute for Science and Education, Oak Ridge, Tennessee, USA; 3 Geospatial Research, Analysis, and Services Program, Office of Innovation and Analytics, Centers for Disease Control and Prevention, Atlanta, Georgia, USA; 4 Booz Allen Hamilton, Atlanta, Georgia, USA; 5 California Emerging Infections Program, Oakland, California, USA

**Keywords:** Foodborne outbreak, Ground beef, Salmonellosis, Social vulnerability

## Abstract

*Salmonella* is estimated to be the leading bacterial cause of U.S. domestically acquired foodborne illness. Large outbreaks of *Salmonella* attributed to ground beef have been reported in recent years. The demographic and sociodemographic characteristics of infected individuals linked to these outbreaks are poorly understood. We employed a retrospective case-control design; case-patients were people with laboratory-confirmed *Salmonella* infections linked to ground beef-associated outbreaks between 2012 and 2019, and controls were respondents to the 2018–2019 FoodNet Population Survey who reported eating ground beef and denied recent gastrointestinal illness. We used county-level CDC/ATSDR Social Vulnerability Index (SVI) to compare case-patient and controls. Case-patient status was regressed on county-level social vulnerability and individual-level demographic characteristics. We identified 376 case-patients and 1,321 controls in the FoodNet sites. Being a case-patient was associated with increased overall county-level social vulnerability (OR: 1.21 [95% CI: 1.07–1.36]) and socioeconomic vulnerability (OR: 1.24 [1.05–1.47]) when adjusted for individual-level demographics. Case-patient status was not strongly associated with the other SVI themes of household composition and disability, minority status and language, and housing type and transportation. Data on individual-level factors such as income, poverty, unemployment, and education could facilitate further analyses to understand this relationship.

The Centers for Disease Control and Prevention (CDC) identifies nontyphoidal *Salmonella* (NTS) enterica as one of the top five pathogens contributing to foodborne illnesses in the United States, responsible for an estimated 1.35 million illnesses per year (Collier et al., 2021; Scallan et al., 2011). Beef is one of the top six food categories commonly identified as the source of *Salmonella* illnesses, behind chicken, fruits, pork, seeded vegetables, and other produce (Interagency Food Safety Analytics Collaboration, 2017, 2018a, 2018b, 2018c, 2019, 2020, 2021). More specifically, ground beef has been the type of beef identified as the source of most outbreaks linked to beef in recent years (Laufer et al., 2015; Richardson et al., 2021); during 2012–2019, 50% of outbreaks linked to a known beef type were linked to ground beef (Canning et al., 2023). Moreover, the number of *Salmonella* outbreaks linked to ground beef remains stable which warrants further research into prevention strategies.

Community-level factors that may contribute to foodborne illness inequities are poorly understood. Although previous studies have documented unequal access to healthy food in neighborhoods by race/ethnicity, socioeconomic status, and urban/rural residency (Zenk et al., 2014; Kern et al., 2017; Ohri-Vachaspati et al., 2019), few have examined access to uncontaminated food or levels of microbial contamination of foods by neighborhood (Quinlan, 2013). Further, none have systematically examined indicators of community-level social vulnerability among people associated with outbreaks linked to contaminated food, nor outbreaks specifically linked to ground beef. Community-level social vulnerability, which can include measures of the negative effects on human health caused by external stresses, can contribute to health inequity. The OASH Office of Disease Prevention and Health Promotion in their Healthy People 2030 (HP2030) Framework outlines health equity as an overarching goal (Office of Disease Prevention and Health Promotion, 2020a). Further, specific objectives relating to the reduction of infections caused by *Salmonella* are listed, and the reduction of outbreaks of *Salmonella* and other enteric bacterial infections associated with beef is under development. However, health equity-specific goals have not previously been included in objectives relating to foodborne illness generally, or ground beef specifically (Office of Disease Prevention and Health Promotion, 2020b).

Understanding who becomes ill during foodborne outbreaks by characterizing both individual patient demographic characteristics and community-based factors, such as social vulnerability, is important to both identify and address potential health inequities in foodborne illnesses and develop effective and tailored prevention strategies. We aimed to characterize possible inequities related to *Salmonella* outbreaks associated with ground beef by comparing both characteristics of populations experiencing inequities (markers, e.g., race/ethnicity) and factors that cause or perpetuate the inequities (drivers, e.g., social vulnerability) among people linked to *Salmonella* outbreaks associated with ground beef, with people who eat ground beef but denied gastrointestinal illness.

## Materials and methods

### Data sources

#### Case-Patients.

CDC’s Foodborne Disease Outbreak Surveillance System (FDOSS) (U.S. Centers for Disease Control and Prevention, 2018) collects information from state and local health departments about foodborne disease outbreaks, which are defined as at least two individuals becoming ill from consuming the same type of contaminated food or drink. Outbreaks are detected using pulsed-field gel electrophoresis or whole–genome sequencing of bacterial isolates from ill people reported to PulseNet, CDC’s national molecular subtyping network for foodborne disease surveillance. PulseNet transitioned from using pulsed-field gel electrophoresis to whole genome sequencing to detect outbreaks in recent years (U.S. Centers for Disease Control and Prevention, 2019). Information collected in FDOSS includes date and location of outbreaks, number of ill individuals, food or drink implicated, setting where implicated food/drink were prepared and eaten, and the pathogen that caused the outbreak. We queried FDOSS to identify all outbreaks fulfilling three criteria: a laboratory-confirmed etiology of *Salmonella*, ground beef listed as the single contaminated ingredient or implicated food, and the first reported illness onset date occurring between 1 January 2012 and 31 December 2019. Case-patients in outbreaks were defined as illness in a person linked to an outbreak (U.S. Centers for Disease Control and Prevention, 2023a). CDC investigators utilize multiple tools to help them identify the source of outbreaks (U.S. Centers for Disease Control and Prevention, 2022). Patient demographic characteristics (age, sex, race, and ethnicity) and patient residence (county) were obtained from PulseNet (U.S. Centers for Disease Control and Prevention, 2021a). To verify that the county reported to PulseNet represented the patient’s county of residence rather than where testing occurred, and to collect race and ethnicity data, state health departments were queried for outbreaks occurring during 2012–2018. Data received from the state health departments classified race into five broad categories (American Indian or Alaska Native, Asian, Black or African American, Native Hawaiian/Pacific Islander, Others, and White). We combined race and ethnicity into a single variable. We first classified patients of any race with Hispanic or Latino ethnicity as Hispanic (any race). We classified patients of non-Hispanic (NH) ethnicity as NH Black or NH White. Patients who identified with NH ethnicity and either multiple races or another race (including American Indian or Alaska Native, Asian, Native Hawaiian/Pacific Islander, and Others) were classified as individuals of NH other race(s) due to the small sample size.

#### Controls.

The Foodborne Diseases Active Surveillance Network (FoodNet) (U.S. Centers for Disease Control and Prevention, 2021b) conducts surveillance for infections commonly transmitted through food. The FoodNet surveillance area includes Connecticut, Georgia, Maryland, Minnesota, New Mexico, Oregon, Tennessee, and selected counties in California, Colorado, and New York – a geographic area that includes approximately 15% of the US population. The FoodNet Population Survey is a periodic survey of randomly selected residents in the FoodNet surveillance area that aims to estimate the disease burden from acute diarrheal illness and the frequency of exposures linked to diarrheal illness (Scallan et al., 2011; Henao et al., 2015). The 2018–2019 FoodNet Population survey was conducted from December 2017 through July 2019. For this analysis, controls were selected among participants in the 2018–2019 FoodNet Population Survey who reported consuming ground beef in the 7 days prior to completing the survey and reported no acute gastrointestinal illness (diarrhea or vomiting) in the 30 days prior to completing the survey. We defined ground beef consumption as eating ground beef prepared either at home or outside the home in the past 7 days, ground beef that was undercooked or raw in the past 7 days, or preformed hamburger patties at home.

#### Social Vulnerability Index (SVI).

**Figure 1.**
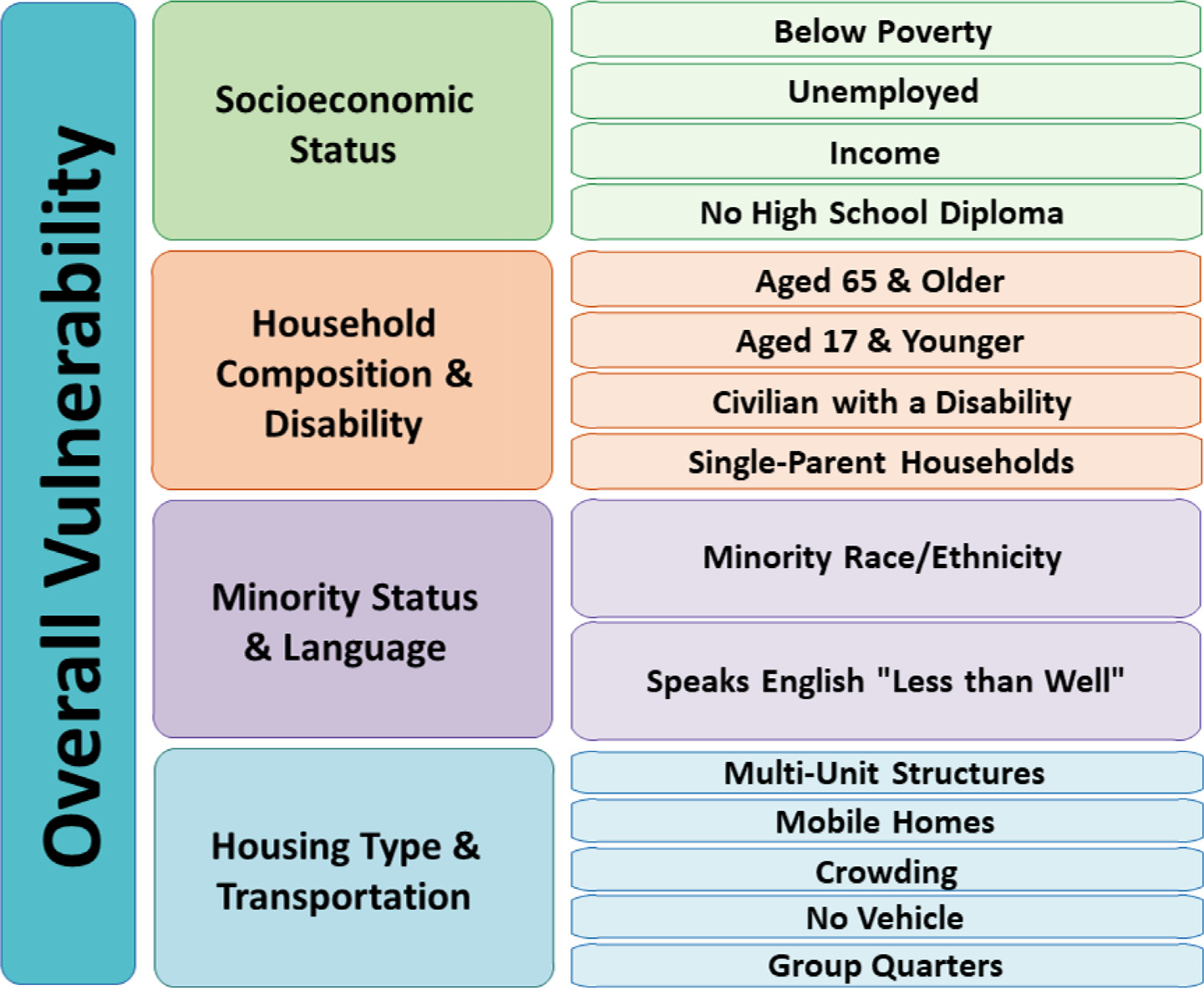
Centers for Disease Control and Prevention/Agency for Toxic Substances and Disease Registry (CDC/ATSDR), Social Vulnerability Index themes and variables.

The Centers for Disease Control and Prevention/Agency for Toxic Substances and Disease Registry (CDC/ATSDR) Social Vulnerability Index (SVI) (U.S. Centers for Disease Control and Prevention/Agency for Toxic Substances and Disease Registry, 2023a) was originally designed to support response efforts in public health emergencies (Flanagan et al., 2018). It has since been used in a variety of research applications, including COVID-19 vaccination coverage (Barry et al., 2021), hurricane impact assessment (Rickless et al., 2021), and heat-related health outcomes (Lehnert, et al., 2020). CDC defines social vulnerability as the potential negative effects on communities caused by external stresses on human health (U.S. Centers for Disease Control and Prevention/Agency for Toxic Substances and Disease Registry, 2023b). The CDC/ATSDR SVI is a quantitative measure of community-level social vulnerability derived from fifteen variables from 5-Year Estimates of the American Community Survey (2014–2018 American Community Survey 5-Year Estimates Database). The variables are grouped into four themes: Socioeconomic Status, Household Composition and Disability, Minority Status and Language, and Housing Type and Transportation (Fig. 1). Each county receives a percentile rank for each theme, and an overall percentile rank combining all the themes. A higher percentile rank corresponds with greater social vulnerability. The original CDC/ATSDR was created in 2011 and updated in 2014, 2016, and 2018. For this study, we matched controls with the 2018 iteration, and matched case-patients with the most recent iteration that corresponded to the year of illness onset of each case-patient.

### Statistical analysis

We used a retrospective case-control design. We selected case-patients and controls who resided in a FoodNet site and with a known county of residence. Probability proportional to size of survey weights (PPS) was applied for sampling controls. The individual standardized survey weights in the 2018–2019 FoodNet Population Survey were used as the size measure to select the final sample of controls by FoodNet site (Devine, manuscript submitted for publication). A ratio of one case-patient to four controls was used to select controls within the same FoodNet sites as the corresponding case-patients. A ratio of one case-patient to three controls was applied for California due to a high number of case-patients relative to a limited number of controls. Using county of residence, we assigned county-level SVI ranks (Centers for Disease Control and Prevention/Agency for Toxic Substances and Disease Registry (CDC/ATSDR), 2022) to individual case-patients and controls. We further excluded case-patients (*n* = 2) and controls (*n* = 22) with unknown sex.

We examined and compared individual patient demographic characteristics and county-level SVI ranks of case-patients and controls using descriptive statistics. For continuous variables (SVI percentile rank and age), the median and interquartile range (IQR) were calculated due to skewness and case-patients were compared to controls using the Wilcoxon Rank-Sum test, while for categorical variables (sex and race/ethnicity), frequencies were calculated and distributions of case-patients were compared to controls using the Pearson Chi–Squared test.

To assess the relationship between case-patient status and county-level SVI ranks, case-patient status was regressed on SVI ranks using marginal models. Specifically, generalized estimating equations (GEEs), specified as binomial distributions with logit link functions, were employed to account for any correlation within FoodNet site. Four models were employed. The first estimated the odds of being a case-patient given the overall SVI; the second replaced the overall SVI with the four SVI themes as predictors. The third and fourth models added age, sex, and race/ethnicity as predictors, to represent individual-level risk factors for ground beef-associated *Salmonella* infection. The odds ratios (OR), unadjusted and adjusted, and 95% confidence intervals (CIs) were estimated for each predictor as the change in odds of being a case-patient associated with changes in predictors (SVI ranks, overall and for each theme in models 1 and 2, plus age, sex, and race/ethnicity in models 3 and 4). All statistical analyses were conducted using SAS version 9.4 (SAS Institute Inc., Cary, NC, USA.).

## Results

### Description of case-patients and controls

During 2012–2019, 798 persons were infected with *Salmonella* and linked to 12 ground beef-associated outbreaks in the United States. Among these, 772 (97%) case-patients reported county of residence, of which 376 (49%) lived within a FoodNet site (including case-patients from nine outbreaks). Among 39,748 respondents that completed the 2018–2019 FoodNet Population Survey, 12,205 (31%) reported ground beef consumption. Among these 12,205 respondents, 9,918 (81%) denied acute gastrointestinal illness; 7,295 (74%) of these respondents reported their county of residence (Supplemental Table 1). After sampling for controls, a total of 376 (22%) case-patients and 1,321 (78%) controls comprised the final analytic data set. Nearly half of the final set of case-patients, 183 (49%), was comprised of people who resided in California; ~50% of FoodNet sites reported <5 case-patients during the study period (Table 1).

**Figure 2.**
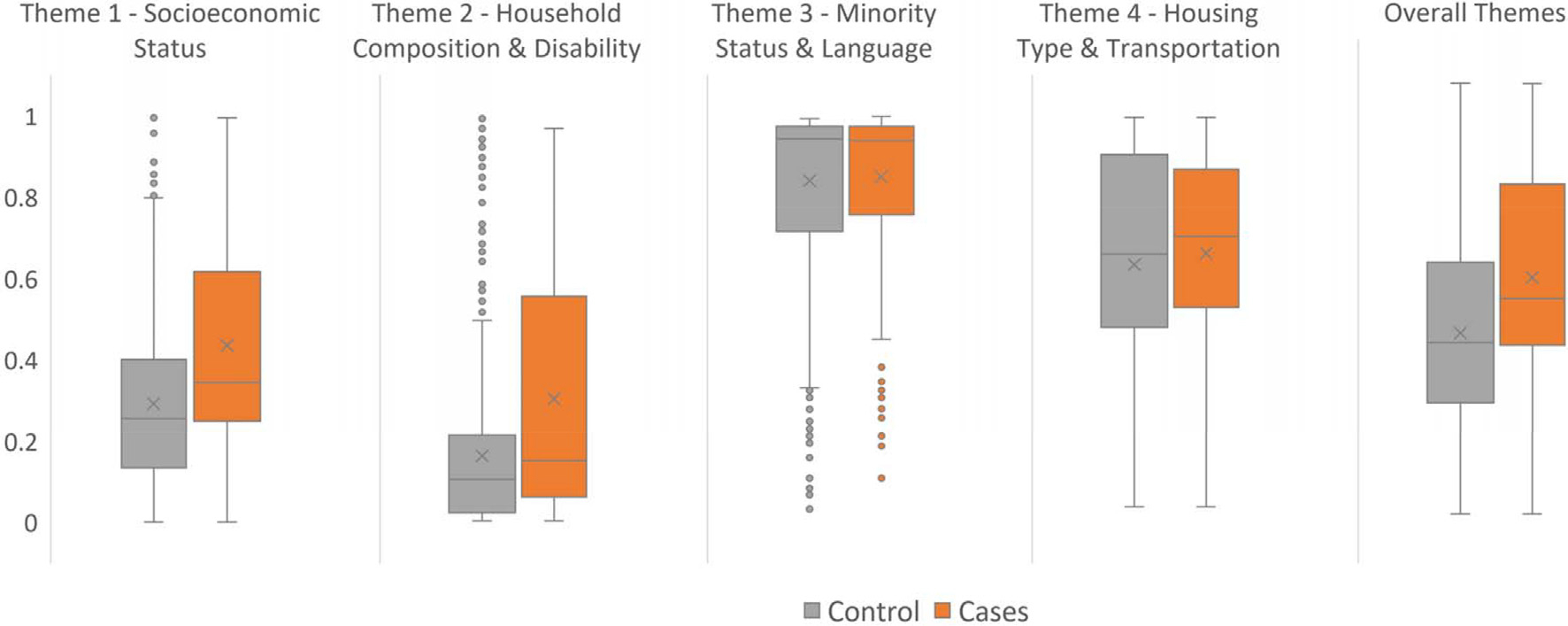
Boxplot ^*a*^ Comparing the Mean of Overall and the Four Themes of Social Vulnerability Index^*b*^ between Case-Patients: Salmonellosis Cases Linked to Ground Beef Associated Outbreaks – CDC’s Foodborne Disease Outbreak Surveillance System (FDOSS) (2012–2019) and Controls: Participants in FoodNet Population Survey (2018–2019) who reported eating ground beef and denied gastrointestinal illness, United States. ^*a*^ Whiskers depict variability outside the lower and upper quartiles, x inside the box depicts the mean, while the line inside the box depicts the median. Outliers are presented as small bubbles outside the whiskers. ^*b*^ Controls were matched with the 2018 SVI, while case-patients were matched with the most recent iteration that corresponded to the year of illness onset of the case. 2018 SVI percentile ranks for Rio Arriba County, NM, were not available, instead, they were replaced by 2016 SVI percentile rank. Higher SVI percentile ranks indicate high social vulnerability, and lower SVI percentile ranks indicate low social vulnerability. SVI percentile rank of 0.5 is considered moderate.

Both case-patients and controls resided in counties with lower vulnerability with respect to socioeconomic status (percentile rank among case-patients = 0.34, controls = 0.25) and household composition and disability (percentile rank among case-patients = 0.15, controls = 0.10) compared with more moderately vulnerable US counties (percentile rank among all US counties = 0.50), but higher vulnerability with respect to minority status and language (percentile rank among case-patients = 0.94, controls = 0.94), and housing type and transportation (percentile rank among case-patients = 0.70, controls = 0.66) (Table 2 and Fig. 2) (U.S. Census Bureau, 2020). Case-patients resided in counties with higher vulnerability compared to controls with respect to socioeconomic status [0.34 (0.25–0.62) vs. 0.25 (0.13–0.40), *p* < 0.001, respectively], and household composition and disability [0.15 (0.06–0.56) vs. 0.10 (0.02–0.21), *p* < 0.001, respectively] (Table 2 and Fig. 2). Similar SVI ranks were observed for case-patients and controls for minority status and language, and housing types and transportation (Fig. 1). Additionally, case-patients resided in counties with higher overall social vulnerability compared with controls [0.51 (0.40–0.77) vs. 0.41 (0.27–0.59), *p* < 0.001 (Table 2 and Fig. 2).

There was a higher proportion of females than males among case-patients (52%) compared to controls (45%), *p* = 0.026 (Table 2). Most case-patients (37%) and controls (35%) were in the 35–64 year age group. A lower proportion of case-patients were aged 5–17 years (14%) compared to controls (22%), while a higher proportion of case-patients were aged 18–34 years compared to controls, 22% vs. 13%, respectively, *p* < 0.001. Most case-patients (60%) and controls (68%) were non-Hispanic White, while a higher proportion of case-patients identified as non-Hispanic other or multiple races (22%) compared to controls (15%), *p* = 0.005 (Table 2).

#### Bivariate and multivariate analyses.

**Figure 3.**
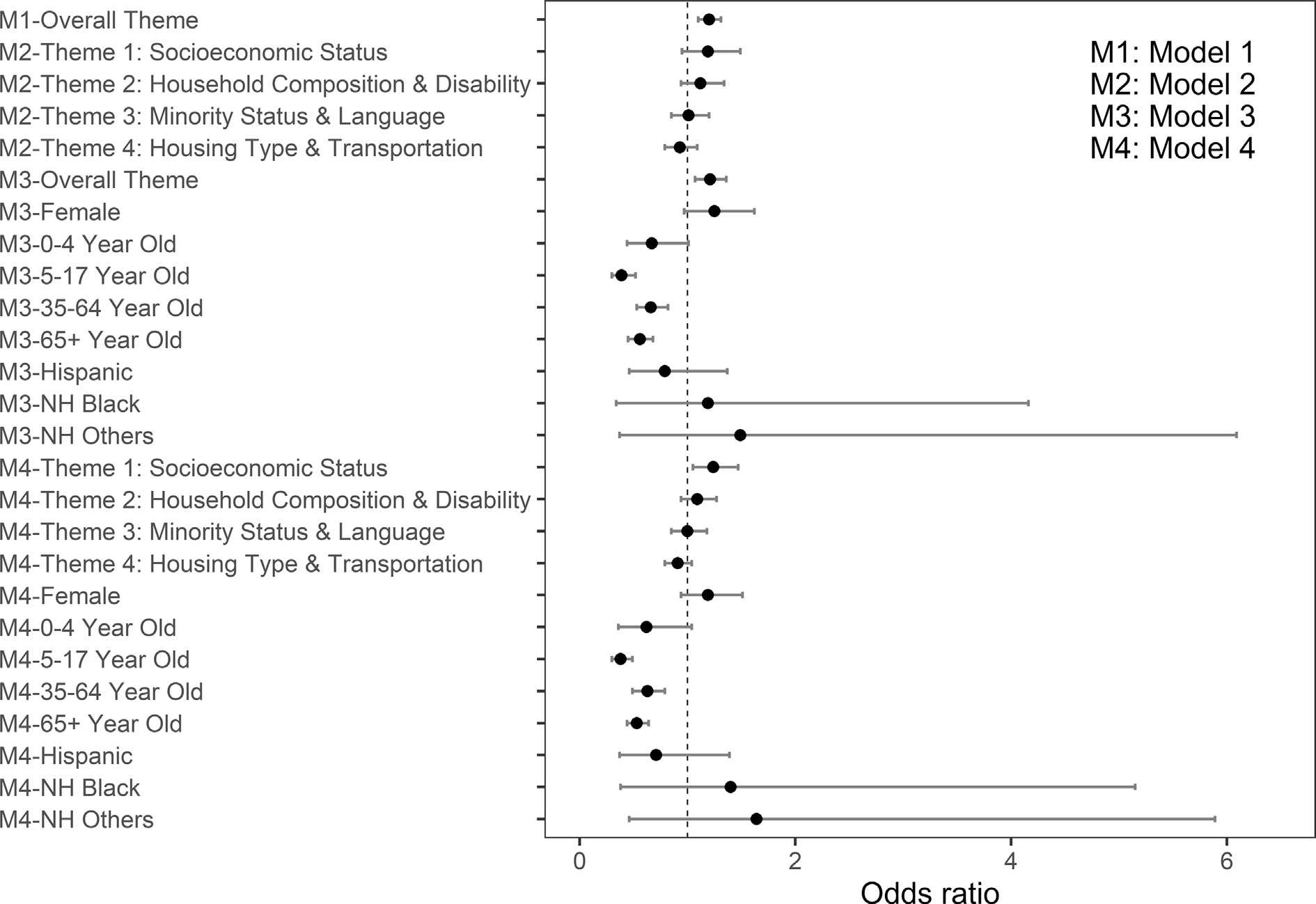
Odds Ratio (OR) and 95% Confidence Intervals (CI) of Case-Patient Status from Logistic Regression for all Four Models^*a*^. Case-Patients: Salmonellosis Cases Linked to Ground Beef Associated Outbreaks – CDC’s Foodborne Disease Outbreak Surveillance System (FDOSS) (2012–2019) and Controls: Participants in FoodNet Population Survey (2018–2019) who reported eating ground beef and denied gastrointestinal illness, United States. ^*a*^ Model (1): modeling case-patient status with overall Social Vulnerability Index (SVI). Model (2): modeling case-patient status with all four SVI themes. Model (3): modeling case-patient status with the overall SVI, adjusted for individual-level sex, age group, and race/ethnicity. Model (4) modeling case-patient status with all four SVI themes, adjusted for individual-level sex, age group, and race/ethnicity. Controls were matched with the 2018 SVI, while case-patients were matched with the most recent iteration that corresponded to the year of illness onset of the case. 2018 SVI percentile ranks for Rio Arriba County, NM, were not available, instead, they were replaced by the 2016 SVI percentile rank.

The crude odds of being a case-patient in Model 1 (overall SVI), (Table 3 and Fig. 3) was positively associated with residing in a county with a higher overall SVI rank, (OR = 1.20, 95% confidence interval [CI] 1.10–1.31, *p* < 0.001), indicating 20% (10–31%) increase in odds of being a case-patient with each 10-point percentile increase in social vulnerability rank. In Model 2, (four SVI themes), the crude odds of being a case-patient was not associated with any of the four themes. The four SVI themes were confirmed to be independent and not strongly correlated with each other using Pearson’s correlation test.

In Models 3 and 4, we adjusted the estimates by including additional covariates: individual-level sex, age, and race/ethnicity. In model 3, (overall SVI adjusting for demographic characteristics), the adjusted odds of being a case-patient increased by 21% (7–36%) with each 10-point percentile increase in overall SVI rank (aOR = 1.21; 95% CI 1.07–1.36, *p* = 0.002). Case-patient status was not associated with sex or race/ethnicity. All age groups had lower odds of being a case-patient compared to those aged 18–34 years (all <0.001), except for children aged 0–4 (aOR = 0.67; 95% CI 0.44–1.01, *p* = 0.055).

In model 4, (four SVI themes adjusting for demographic characteristics), the odds of being a case-patient increased by 24% (5–47%) with each 10-point percentile increase in socioeconomic status vulnerability rank (aOR = 1.24, 95% CI: 1.05–1.47, *p* = 0.010). Similar to Model 2 (four SVI themes), which did not adjust for demographic characteristics, case-patient status was not associated with household composition and disability theme, minority status and language theme, or housing type and transportation theme. Similar to model 3 (overall SVI adjusting for demographic characteristics), case-patient status was not associated with sex or race/ethnicity. Case-patient status was associated with age; all age groups had lower odds of being a case-patient compared to those aged 18–34 years, except for children aged 0–4 years (aOR = 0.62, 95% CI: 0.36–1.04, *p* = 0.0.72) (Table 3 and Fig. 3) (U.S. Census Bureau, 2020).

## Discussion

Our findings identify possible health inequities among those linked to a *Salmonella* outbreak associated with ground beef compared with nonill ground beef eaters. In this case-control analysis, we found that after adjusting for other SVI themes and demographic characteristics, the odds of being linked to a ground beef-associated outbreak increased by 24% (5–47%) with each 10-point percentile increase in county-level socioeconomic status vulnerability rank. Further, the odds of being linked to a ground beef-associated outbreak increased by 21% (7–36%) with each 10-point percentile increase in overall county-level SVI rank. Both case-patients and controls lived in counties with similarly relatively high vulnerability with respect to minority status and language theme (0.94 and 0.94, respectively) and housing type and transportation theme (0.70 and 0.66, respectively) when compared to the nationwide median for US counties (0.50). These findings suggest that community-level factors, such as socioeconomic status, might be markers of risk for ground-beef-associated salmonellosis, and if such a relationship is confirmed in future analyses, it could help identify communities at higher risk for *Salmonella* infections linked to ground beef, and inform community-based intervention strategies to prevent these infections.

Although both case-patients and controls lived in counties with relatively low social vulnerability, evidenced by SVI percentile rank scores under the nationwide median, case-patients had increased odds of living in a county that was more vulnerable with respect to socioeconomic status, compared with controls. However, in another analysis (Waltenburg, manuscript submitted for publication), when case-patients were compared with the general population, the SVI socioeconomic status theme was not significantly different. There are several factors related to socioeconomic status that could explain our finding in this analysis, including systematic differences in the ground beef that is purchased and consumed, or in storage, handling, and preparation of food containing ground beef. Those residing in communities with higher socioeconomic vulnerability may have less access to uncontaminated ground beef. A study conducted in the Philadelphia metropolitan area identified a difference in the microbial quantity of produce at markets by socioeconomic status of the census tract of the market locations (Koro et al., 2010). Our findings might also reflect systematic differences at the community level in access to and condition of appliances to store and cook ground beef, access to food thermometers, and access to food safety information for both retailers (e.g., grocery stores, markets, restaurants) and consumers (Oakley et al., 2019). However, further research is needed to identify and better understand whether these or other specific factors that may be related to socioeconomic status at both the individual and community levels and ultimately contribute to the risk of salmonellosis from ground beef consumption.

Among people who ate ground beef, case-patients and controls lived in counties with similarly high vulnerability for the minority status and language SVI theme, which may reflect the general demographics of people who eat ground beef. In another study that compared ill people linked to ground beef-associated *Salmonella* outbreaks with the general population, which includes people who don’t eat ground beef, a higher proportion of non-Hispanic White persons and non-Hispanic American Indian or Alaska Native persons was found among ill people involved in *Salmonella* outbreaks associated with ground beef (Waltenburg, manuscript submitted for publication). Among people who ate ground beef, we found that race and ethnicity were not associated with illness in adjusted models. However, it is possible that we did not detect any differences between controls and case-patients regarding race and ethnicity in this analysis because we were only able to analyze race and ethnicity using four broad categories due to the small sample size. Of note, though not statistically significant, the proportion of case-patients who were non-Hispanic White was slightly lower compared to controls (60% vs. 68%), and the proportion of case-patients who were categorized as non-Hispanic other or multiple races, which would include non-Hispanic American Indian or Alaska Native persons, was higher compared with controls (22% vs. 15%).

In each adjusted model (models 3 and 4), the 18–34 years age group had higher odds of being ill due to a *Salmonella* outbreak associated with ground beef when compared to all other age groups. One potential explanation of this finding could be related to consumer preferences for undercooked ground beef or practices around ground beef preparation. Among respondents of the 2018–2019 FoodNet Population Survey, the 18–34-years age group reported more frequent consumption of undercooked or raw ground beef than all other ages, and the same age group reported the lowest ownership of food thermometers compared with all other age groups (U.S. Centers for Disease Control and Prevention, 2023b).

The results of this analysis are subject to at least seven limitations. First, the number of reported case-patient is likely an underestimate of all case-patient of *Salmonella* linked to ground beef, as not all ill persons seek medical care and are tested for *Salmonella*. In fact, it is estimated that only 1 in 29 case-patient is tested (Scallan et al., 2011). Therefore, the differences in who seeks medical care and is tested could affect our ability to understand inequities in disease occurrence. Further, illnesses linked to outbreaks represent a small proportion of all *Salmonella* illnesses (Collier et al., 2021). Second, despite efforts to obtain more complete race/ethnicity data, 21% of these data were missing for case-patients, which could leave gaps in our results. Third, controls were selected from the 2018–2019 FoodNet Population Survey, and case-patient were selected from the FoodNet surveillance area, which may not be representative of the United States population for several reasons: it underrepresents Hispanic persons compared with the total US population, the survey was administered in English and Spanish only and the control group may exclude persons who exclusively spoke other languages, and the SVI of counties of FoodNet Population Survey participants is skewed compared to more moderately vulnerable US counties. Fourth, over 49% of case-patients and 43% of the final analytic sample resided in CA; therefore, our findings may not be representative at the national level. Fifth, controls from the 2018–2019 FoodNet Population Survey self–reported ground beef consumption and health conditions and may be subject to recall bias. Sixth, as with all geographically aggregated demographic data, it is important to note that a large variation of individual-level vulnerability exist within resident of a single county population. Finally, in our analysis, we compared case-patients from 2012 to 2019 to controls from the 2018–2019 FoodNet Population Survey; although very unlikely, an individual could have been included both as case-patient and as a control, and our analysis did not take into consideration the effect on the change of food trends. However, when case-patient from only 2015–2019 outbreaks were included, the results of the four models were very similar.

The findings from this analysis help identify possible health inequities in *Salmonella* infections among people who eat ground beef and suggest that community-level factors like socioeconomic status are associated with increased risk of illness. Analyzing community-level social factors using tools like CDC/ATSDR’s SVI can help characterize underlying social determinants of illness risk among people linked to outbreaks, particularly when individual-level data are not available. While many interventions to reduce foodborne illness focus on how individual consumers store, handle, and prepare ground beef, community-level differences in conditions in which persons live, work, play, and access care highlight challenges to successfully implementing them and emphasize the importance of improving the safety of ground beef before it reaches consumers. Collecting more robust and complete individual-level demographic and social data and routinely analyzing it alongside community-level social factors can help inform and focus prevention strategies to reduce health inequities.

## Supplementary Material

Supplementary table 1

## Figures and Tables

**Table 1:** Number of available and selected controls - Participants in FoodNet Population Survey (2018–2019) who reported eating ground beef and denied gastrointestinal illness, and Salmonellosis Cases Linked to Ground Beef Associated Outbreaks^a^ — CDC’s Foodborne Disease Outbreak Surveillance System (FDOSS) (2012–2019) by FoodNet site

**FoodNet Site**	**Controls (All)**^b^ 7,295	**Controls (Selected)** 1,321 (78%)	**Case-patients**^c^ 376 (22%)	**Final Sample Size** 1,697 (100%)

California ^d^	789	549	183	732
Colorado ^d^	664	316	79	395
Connecticut	700	8	2	10
Georgia	762	16	4	20
Maryland	658	8	2	10
Minnesota	807	168	42	210
New Mexico	760	144	36	180
New York ^d^	714	92	23	115
Oregon	677	4	1	5
Tennessee	764	16	4	20

a Alameda, Contra Costa, and San Francisco counties are included in the FoodNet surveillance area. The 2018–2019 FoodNet Population Survey was also conducted in Los Angeles County. Case-patients and controls in this study were selected from both the FoodNet surveillance area and Los Angeles County.

b Total number of available controls after applying the inclusion and exclusion criteria.

c Number of outbreak cases for these states: CA: 3; CO: 3; CT: 2; GA: 2; MD: 2; MN: 5; NM: 3; NY: 3; OR: 1; TN: 2 (See Supplemental Table 1 for more details).

d These sites do not include all counties.

**Table 2:** Descriptive statistics for the county-level Social Vulnerability Index^a^ (SVI) percentile ranks (2010–2018)^b^ and individual-level demographics of Salmonellosis Cases Linked to Ground Beef Associated Outbreaks — CDC’s Foodborne Disease Outbreak Surveillance System (FDOSS) (2012–2019) and controls - participants in FoodNet Population Survey (2018–2019) who reported eating ground beef and denied gastrointestinal illness

	Final Sample Size (*n* = 1697)	Controls (*n* = 1321)	Case-Patients (*n* = 376)	*p value* ^c^

***SVI Percentile Ranks**, median (IQR)*				
Theme 1: Socioeconomic Status	0.25 (0.17–0.52)	0.25 (0.13–0.40)	0.34 (0.25–0.62)	<0.001
Theme 2: Household Composition & Disability	0.10 (0.02–0.29)	0.10 (0.02–0.21)	0.15 (0.06–0.56)	<0.001
Theme 3: Minority Status & Language	0.94 (0.72–0.97)	0.94 (0.72–0.97)	0.94 (0.76–0.97)	0.698
Theme 4: Housing Type & Transportation	0.66 (0.48–0.90)	0.66 (0.48–0.90)	0.70 (0.53–0.86)	0.630
Overall SVI	0.42 (0.31–0.68)	0.41 (0.27–0.59)	0.51 (0.40–0.77)	<0.001
***Demographics**, n (%)*				
Sex				0.026
Male	902 (53)	722 (55)	180 (48)	
Female	793 (47)	599 (45)	194 (52)	
Age group, in years				<0.001
0–4	79 (5)	61 (5)	18 (5)	
5–17	335 (20)	284 (22)	51 (14)	
18–34	256 (15)	175 (13)	81 (22)	
35–64	603 (35)	465 (35)	138 (37)	
65 +	420 (25)	336 (25)	84 (23)	
Race/ethnicity				0.005
Hispanic or Latino	235 (14)	183 (14)	52 (14)	
Non-Hispanic Black	52 (3)	38 (3)	14 (4)	
Non-Hispanic White	1131 (67)	904 (68)	227 (60)	
Non-Hispanic other or multiple race	279 (16)	196 (15)	83 (22)	

a Higher SVI percentile ranks indicate high social vulnerability, and a lower SVI percentile rank indicates low social vulnerability. SVI percentile rank of 0.5 is considered moderate.

b 2018 SVI percentile ranks for Rio Arriba County, NM, were not available, instead they were replaced by 2016 SVI percentile rank. The underlying data for the 2018 SVI is 2014–2018 ACS estimates. Controls were matched with the 2018 SVI, while case-patients were matched with the most recent iteration that corresponded to the year of illness onset of the case.

c Wilcoxon Rank test was used for comparison of median distribution (SVI ranks), and Chi sq test of proportion was used to compare demographic indicators.

**Table 3:** Odds Ratio (OR) of Case-Patient Status from Logistic Regression. Case-Patients: Salmonellosis Linked to Ground Beef Associated Outbreaks — CDC’s Foodborne Disease Outbreak Surveillance System (FDOSS) (2012–2019) and Controls: Participants in FoodNet Population Survey (2018–2019) who reported eating ground beef and denied gastrointestinal illness, United States

	Model 1^a^		Model 2^a^		Model 3^a^		Model 4^a^	
	Unadjusted OR (95% CI)	*p value*	Unadjusted OR (95% CI)	*p value*	Adjusted ^c^ OR (95% CI)	p value	Adjusted ^c^ OR (95% CI)	*p value*

Overall Theme ^b^	1.20 (1.10–1.31)	**<0.001**			1.21 (1.07–1.36)	**0.002**		
Theme 1: Socioeconomic Status ^b^			1.19 (0.95–1.49)	0.120			1.24 (1.05–1.47)	**0.010**
Theme 2: Household Composition & Disability^b^			1.12 (0.94–1.34)	0.198			1.09 (0.94–1.27)	0.252
Theme 3: Minority Status & Language ^b^			1.01 (0.85–1.20)	0.893			1.00 (0.85–1.18)	0.983
Theme 4: Housing Type & Transportation ^b^			0.93 (0.79–1.09)	0.357			0.91 (0.79–1.04)	0.156
Sex								
Male					Ref	Ref	Ref	Ref
Female					1.25 (0.97–1.62)	0.090	1.19 (0.94–1.51)	0.153
Age group, in years								
0–4					0.67 (0.44–1.01)	0.055	0.62 (0.36–1.04)	0.072
5–17					0.39 (0.30–0.52)	**<0.001**	0.38 (0.30–0.49)	**<0.001**
18–34					Ref	Ref	Ref	Ref
35–64					0.66 (0.53–0.82)	**<0.001**	0.63 (0.49–0.79)	**<0.001**
65 +					0.56 (0.45–0.68)	**<0.001**	0.53 (0.44–0.64)	**<0.001**
Race/ethnicity								
Hispanic or Latino					0.79 (0.46–1.37)	0.402	0.71 (0.37–1.39)	0.319
Non-Hispanic Black					1.19 (0.34–4.16)	0.786	1.40 (0.38–5.15)	0.615
Non-Hispanic White					Ref	Ref	Ref	Ref
Non-Hispanic others multiple race					1.49 (0.37–6.09)	0.575	1.64 (0.46–5.89)	0.448

a Model (1): modeling case-patient status with overall Social Vulnerability Index (SVI). Model (2): modeling case-patient status with all four SVI themes. Model (3): modeling case-patient status with the overall SVI, adjusted for individual-level sex, age group, and race/ethnicity. Model (4) modeling case-patient status with all four SVI themes, adjusted for individual-level sex, age group, and race/ethnicity. Controls were matched with the 2018 SVI, while case-patients were matched with the most recent iteration that corresponded to the year of illness onset of the case.

b Odds of case-patient status associated with a 10-point percentile increase in SVI percentile ranks. Controls were matched with the 2018 SVI, while case-patients were matched with the most recent iteration that corresponded to the year of illness onset of the case.

c Adjusted for sex, age group, and race/ethnicity.

## Data Availability

*Salmonella* illness data is available upon request from CDC’s Foodborne Disease Outbreak Surveillance System (https://wwwn.cdc.gov/norsdashboard); some data elements analyzed here may be restricted. CDC’s FoodNet Population Survey data is available (https://www.cdc.gov/foodnet/surveys/population.html). CDC/ATSDR’s SVI data is available (https://www.atsdr.cdc.gov/placeandhealth/svi/index.html).
