# Supplementary material for: Comparing Individual and Community-level Characteristics of People with Ground Beef-associated Salmonellosis and Other Ground Beef Eaters: A Case-control Analysis
Source: J Food Prot. Author manuscript; Available in PMC 2024 Jul 1. (PMC11213658; doi:10.1016/j.jfp.2024.100303)
Supplement: Supplementary table 1 [file NIHMS2000581-supplement-Supplementary_table_1.docx]

**Supplemental Table 1. Ground Beef Associated Outbreaks Salmonellosis Cases Inclusion Criteria; Cases within FoodNet Surveillance Sites*^a^* and Availability of County of Residence - CDC’s Foodborne Disease Outbreak Surveillance System (FDOSS) (2012–2019).**

| Outbreak | Year | Outbreak Size | States involved | Number of cases with known county of residence | Number of cases within FoodNET sites |
| --- | --- | --- | --- | --- | --- |
| 1 | 2012 | 47 | MA, MD, ME, NC, NH, NJ, NY, PA, RI, VT, WV | 47 | 21 |
| 2 | 2012 | 19 | MN | 19 | 19 |
| 3 | 2012 | 18 | AZ, IA, IL, MI, PA, WI | 18 | 0 |
| 4 | 2013 | 39 | GA, IA, IL, MN, NC, SD, WI, WV | 39 | 14 |
| 5 | 2014 | 3 | VA | 3 | 0 |
| 6 | 2014 | 47 | AL, CO, IA, IN, KY, LA, ME, MO, NC, NE, OH, OK, PA, SC, TN, TX, WA, WI, WV | 47 | 15 |
| 7 | 2017 | 106 | AK, AZ, CA, CO, FL, IA, LA, MD, MI, MT, NH, NM, NV, NY, OH, OK, PA, SC, SD, TX, UT, WY | 106 | 42 |
| 8 | 2017 | 5 | MN | 5 | 5 |
| 9 | 2018 | 3 | WI | 3 | 0 |
| 10 | 2018 | 433 | AK, AZ, CA, CO, CT, HI, IA, ID, IL, IN, KS, KY, LA, MA, MN, MO, MS, MT, NM, NV, NY, OH, OK, OR, SD, TN, TX, UT, WA, WV, WY | 433 | 250 |
| 11 | 2019 | 13 | CA, CO, IA, KS, NM, OK, TX, WA | 9 | 3 |
| 12 | 2019 | 48 | CT, GA, IL, KS, KY, MA, ME, MI, MN, NC, NE, OH, TX, VA, WI | 43 | 7 |

*^a^* FoodNet Survillance Sites seven full states (Connecticut, Georgia, Maryland, Minnesota, New Mexico, Oregon, and Tennessee) and specific counties within three other states (California, Colorado, New York), cases from all 10 states were included in this analysis.
